# Resolution of Cox mediated inflammation by Se supplementation in mouse experimental model of colitis

**DOI:** 10.1371/journal.pone.0201356

**Published:** 2018-07-31

**Authors:** Ramanpreet Kaur, Shivani Thakur, Pulkit Rastogi, Naveen Kaushal

**Affiliations:** 1 Department of Biophysics, BMS Block -II, Panjab University, South Campus, Chandigarh, India; 2 Department of Hematology, Post Graduate Institute of Medical Education and Research (PGIMER), Chandigarh, India; Toho Daigaku, JAPAN

## Abstract

UC a form of IBD is a chronic inflammatory disorder of large intestine, with unknown etiology. Reports suggest a critical role of COX-2 dependent prostaglandins (PGs) mediated inflammatory pathway in pathophysiology of UC. However, COX inhibition using NSAIDs exacerbate IBD and thus is not a viable solution. Currently, in DSS induced experimental colitis in mice, we have demonstrated that dietary Se supplementation (0.5ppm as sodium selenite) symptomatically resolves the signs of inflammation in a redox sensitive manner as compared to Se deficient (0.01ppm) conditions, as seen by modulation in oxidative stress markers, morphological changes, histopathological examinations, biochemical studies such as MPO activity, activity of intestinal markers enzymes as well as mRNA and expressions of various pro and anti-inflammatory factors such as, mPGES, hPGDS, TXAS, 15-PGDH, GPX-1 and GPX-2. These findings were validated and correlated with changes in the biophysical parameters such as membrane fluidity, electrical parameters (impedance), transport across the colonic tissue and FTIR. Current study not only concluded that Se at supranutritional concentrations by modulating the redox status relieves the signs of colitis by regulating COX dependent PG biosynthetic pathway, but also sheds light on the biophysical characterization of these inflammatory/resolution pathways involved in UC.

## Introduction

Inflammatory Bowel disease (IBD), mainly comprising of Ulcerative colitis (UC) and Crohn’s disease (CD) is a chronic inflammatory condition of gastrointestinal tract which according to CCFA affects approximately 5 million people worldwide. UC and CD not only affect different locations within gastrointestinal tract, but also have distinct histological inflammatory patterns and many disease specific complications. The onset of inflammatory cascade in intestinal mucosa however, is central to IBD including UC [1].

These inflammatory responses as seen in UC are related to activation of various lipid mediators including prostaglandins (PGs) [2]. Cyclooxygenase (COX) dependent prostaglandin (PG) biosynthesis is central to inflammation and thus is one of the most comprehensively studied pathways in inflammatory conditions [3]. Particularly, the inducible COX-2 isoform involved in various inflammatory reactions leads to exaggerated PGs release [4–7]. This uncontrolled release of PGs by COX-2 can be controlled by Nonsteroidal Anti-Inflammatory Drugs (NSAIDs). However, reports suggest a direct relation between use of NSAIDs and increased UC incidence or more frequent relapse of pre-existing intestinal inflammation [8,9]. These studies suggest that at least in colitis associated inflammation, use of classical anti-inflammatory drugs to inhibit COX is detrimental. Thus, there is demand and need for better modulators of PG biosynthesis pathways, which can aid in the activation of resolution program in UC. Further, increased generation of pro-oxidants in the colonic tissues of UC patients has established the redox basis of UC pathogenesis [10–12].

Pharmacological intervention in human UC patients also attests to the existence of a link between the redox sensitive modulation of PGs metabolism and UC. In this direction, studies with supplementation of dietary nutrients to manipulate exacerbated oxidative stress (OS) in the (dextran sulfate sodium) DSS-induced colitis [13] not only establish the redox basis of UC, but also emphasize the ability of micronutrients to maintain the ambient redox balance to work as therapeutic agents. While, there has been extensive effort to understand the role of inflammatory mediators in UC and their redox regulation, there is little information about this link.

In this direction, studies have shown that Selenium (Se) an essential antioxidant trace element, as anti-inflammatory agent can play a critical role in emendation of inflammatory pathways associated with UC [14–16]. Studies suggest that different doses, duration and forms of Se have varied degree of absorption and bioavailability [17], which might be responsible for its wide spectrum of pathophysiological effects. For example: at its Supra-nutritional doses Se evidently has anti-carcinogenic effects in different types of cancers. In terms of colitis optimum dose of 0.1ppm Se as Sodium Selenite in diet has been proven to reach the adequate Se levels, whereas 0.4ppm Se supplemented in diet, till doses even upto 2μg/kg Body weight have shown to pose protection against the severity of colitis [14][18]. Recently, Zhu *et al* [15] have shown that Se nanoparticles supplemented with 0.8ppm Se resolve the gross signs of inflammation in DSS induced colitis model. Epidemiological evidence also suggests an inverse correlation between UC severity with nutritional Se status [19–22] indicating that malabsorption of nutrients is associated, in part, with epithelial injury and poor resolution of inflammation.

Current study is aimed to understand the plausible modulation of COX dependent inflammatory pathway of mice model of UC by Se in redox sensitive manner. Of importance is an attempt towards biophysical characterization of UC associated inflammation in experimental mice model and its plausible modulation by Se.

## Materials and methods

### Ethics statement

The present study was carried out in accordance with the Committee for the purpose of control and supervision of experimental animals (CPCSEA), Govt. of India guidelines. The Study was approved by Institutional animal ethical committee (IAEC) of Panjab University, Chandigarh with Registration Number PU/IEAC/S/14/20. The mice were housed in polypropylene cages with 12h light and dark cycles in temperature maintained animal rooms in accordance with the guidelines of IAEC and experiments were reviewed and approved before being conducted.

### Animals and experimental design

Male Balb/c mice in the body weight range of 20-25g were procured from the Central Animal House, Panjab University (Chandigarh, India). Animals were acclimatized to departmental animal rooms for a period of 1 week prior to the start of treatment. The animals were randomly segregated in three groups (n = 4–6) and were fed Yeast-based synthetic diets prepared in the laboratory by adding different concentration of Se as Sodium Selenite in Se-Deficient (Se-Def) diet for 8 weeks as reported earlier [23]: (1) Mice kept on yeast based Se-def basal diets contain 0.01 ppm Se, which is below the normal recommended limits were considered as Se deficient. (2) Se was added as sodium selenite in the concentration of 0.1ppm to make the adequate (Se-Ade) group and (3) Se supplemented (Se-Sup) diet was created by adding 0.5 ppm sodium selenite in Se deficient diet for at least 8 weeks. The doses used in the current study are physiologically relevant, well below the toxic levels and have demonstrated beneficial pathophysiological effects. Based on the extrapolation of Se toxicity animal studies the Recommended Dietary Allowances (RDAs) for normal adult is 55μg-200μg per day [24]. Animals were weighed weekly for changes in body weights. All experiments and protocols were approved by the institutional animal ethical committee. After completion of the diet feeding schedule, experimental colitis was induced in mice as described below.

### Induction of colitis

The colitis was induced in Balb/C mice with dextran sodium sulfate (DSS, MW = 40kDa; MP Biomedicals, USA) dissolved in sterile filtered water at a final concentration of 4% (w/v) and presented to mice as drinking water for 5 consecutive days after 8 weeks of Se diet protocol [25]. Fresh DSS solution was provided every day and no change in normal drinking consumption was noted throughout the duration of the study in any of the treatment groups. Following 5 days, the mice were switched on to double distilled water for the remainder of the experiment. Animals were weighed daily and monitored clinically for rectal bleeding, diarrhea, and signs of morbidity. The disease activity index (DAI) was determined by the methods of Tanaka et al, [26] based on sum of scoring trait of stool, and blood in the stool from day 0 to day 10 after colitis induction. Moribund mice or mice that had lost more than 25% of their body weight were sacrificed immediately and listed as dead following induction of DSS colitis as reported earlier [25], and colon lengths were measured during the sample collection. After completion of DSS treatment, animals were sacrificed under ether anesthesia followed by cervical dislocation. Tissues were removed immediately for various analyses.

### Histopathological examination and scoring of colitis

To establish the induction of colitis and ability of Se to ameliorate its effects, the standard established procedures of measuring colon lengths and histopathological scoring [27] are followed.

### Biochemical estimations

#### Preparation of colonic homogenates and brush border membrane (BBM)

Tissue homogenates were prepared (10% w/v) in RIPA buffer using mechanically driven Teflon-fitted potter elvejham-type homogenizer under ice cold conditions followed by preparation of Colonic BBM [28].

#### Se-dependent Glutathione peroxidase (GPx) activity

GPx activity was assayed by the coupled enzyme procedure with glutathione reductase using H_2_O_2_ as substrate [29]. The total protein estimation was performed using the method of Lowry [30].

#### Lipid peroxidation (LPO) and Catalase assay

The levels of LPO were assayed using the method of Wills [31] using 1,1’,3,3’- tetraethoxypropane as standard. Catalase activity was estimated by method of Luck [32].

#### Myeloperoxidase (MPO) activity

Myeloperoxidase activity was measured in the colonic tissues by the method of Bradley [33].

#### Assay of intestinal marker disaccharidases and alkaline phosphatase

The activities of intestinal marker enzymes Sucrase and lactase were determined in homogenates and BBM (brush border membrane) by using a glucose oxidase–peroxidase enzymatic system (GOD-POD) [34]. Intestinal Alkaline phosphatase activity was assayed according to the method of Bergmeyer [35].

### Transport studies

The inflammation induced altered transport of ions or molecules across the membrane of intestine were studied by ring method [36].

#### Amino acid (Histidine) transport and estimation

Transport of histidine along the glucose through the intestinal membrane and an increase in the rate of appearance of histidine from the medium in the lumen of colon is taken as an index of histidine transport across the membrane, which can be estimated spectrophotometrically [37].

### Gene and protein expression analysis

The changes in the gene and protein expression of various inflammatory and anti-inflammatory factors such as GPx 1/2, Cox 1, Cox-2, mPGES, TXAS, hPGDS, 15PGDH and GAPDH were analyzed by semi-quantitative PCR (RT-PCR) and ELISA respectively. For RT-PCR, RNA was isolated using TRI-reagent and the primer sets used are listed in Table 1. The template RNA used for RT-PCR is 1 μg and PCR was done by SuperScript® III One-Step RT-PCR kit (invitrogen). The reaction mixture was incubated at 50°C for 15–30 minutes followed by denaturation at 94°C for 2 minutes. The cDNA so synthesized is further amplified by incubation at different temperatures (94°C for 15 sec, 60°C for 30 sec, 68°C for 1 min/Kb) for 40 cycles. The PCR product was given a final extension by incubating at 68°C for 5 min. The agarose gel electrophoresis was performed to visualize the changes in expression.

**Table 1:** List of primers used.

*Gene*	*Sequence*
**GSH-Px**	Sense 5’-CCTCAAGTACGTCCGACCTG-3’ Antisense 5’-CAATGTCGTTGCGGCACACC-3’
**COX-1**	Sense 5’-GGGAATTTGTGAATGCCACC-3’ Antisense 5’-GGGATAAGGTTGGACCGC-3’
**COX-2**	Sense 5’-AGCGAGGACCTGGGTTCA-3’ Antisense 5’-AAGGCGCAGTTTATGTTGTCTGT-3’
**hPGDS**	Sense 5’-GAATAGAACAAGCTGACTGGC-3’ Antisense 5’-AGCCAAATCTGTGTTTTTGG-3’
**mPGES**	Sense 5’-CCAGATGAGGCTGCGGAAGA-3’ Antisense 5’-AGCGAAGGCGTGGGTGGTTCA-3’
**15-PGDH**	Sense 5’-AAGCTTCTGCACCATGCACGTGA-3’ Antisense 5’-GCGGATCCTTCAGCTATGGCTAAC-3’
**TXAS**	Sense 5’-AGGCTTCTGAAAGAGGTGGACCCT-3’ Antisense 5’-TGAAATCACCATGTCCAGATAC-3’
**GAPDH**	Sense 5’-ATTGTCAGCAATGCATCCTG-3’ Antisense 5’-ATGGACTGTGGTCATGAGCC-3’

For protein expression tissue homogenates are prepared in ice-cold RIPA buffer as described above and protein concentrations were determined in the supernatants using Lowry’s method [30] and 20μg of protein was subjected ELISA as described earlier [38].

### Biophysical analyses

#### Measurement of membrane microviscosity using pyrene as an extrinsic fluorophore

The lateral diffusion of BBM was studied by measuring Pyrene fluorescence excimer (dimer) formation [39] as described earlier [40]. The viscosity/fluidity was calculated from the monomer/excimer fluorescence intensity ratios using following formula:

E/M(excimerfluorescence)/(monomerfluorescence)=(Pyrene)TK/η

Where T is absolute temperature, K is Boltzman constant (1.38062 x 10^−23^ j/k) and η is the microviscosity, while the pyrene concentration was given in 5 mM.

#### Impedance studies

The resistance and capacitance of intestinal tissue was measured at various frequencies (100Hz and 1KHz) for epithelial barrier characterization and transport properties in UC [41]. ~1cm of intestinal tissue from different groups was dehydrated in absolute alcohol (15 minutes) and placed in sample holder of LCR meter containing PBS. The resistance and capacitance of all the tissue samples was measured from 100Hz and 1KHz and the errors are removed by subtracting resistance and capacitance from the PBS measurements. Impedance was calculated from these values using the formula:

$$Impedance\;\left(Z\right)={({\left(R\right)}^{2}+\left(X_{L}‑X_{C}\right))}^{1/2}$$

#### FTIR (Fourier Transform Infrared Spectroscopy)

Colon tissue was lyophilized and homogeneously mixed with KBr (spectroscopic grade) in the ratio of 5:95. The mixture was then pressed at a pressure of 10–15 tones with hydraulic pressure machine and pellets were obtained. FTIR spectra were then recorded in the IR spectrophotometer (Hitachi, Model 270–50) in the range of 900cm^-1^ to 4000cm^-1^. Respective naïve controls (non DSS) from each group were also run simultaneously.

### Data analysis

All data are expressed as mean ± SEM of at least n = 4 independent observations. An unpaired, two-tailed ‘t’ test is used to compare the mean for each treatment group (i.e. Se-Ade and Se-Sup groups) with the mean of the Se Def group. *p* values ≤ 0.05 were considered as statistically significant.

## Results

### Differential Se status resolves signs of inflammation associated with experimental colitis

**Fig 1:**
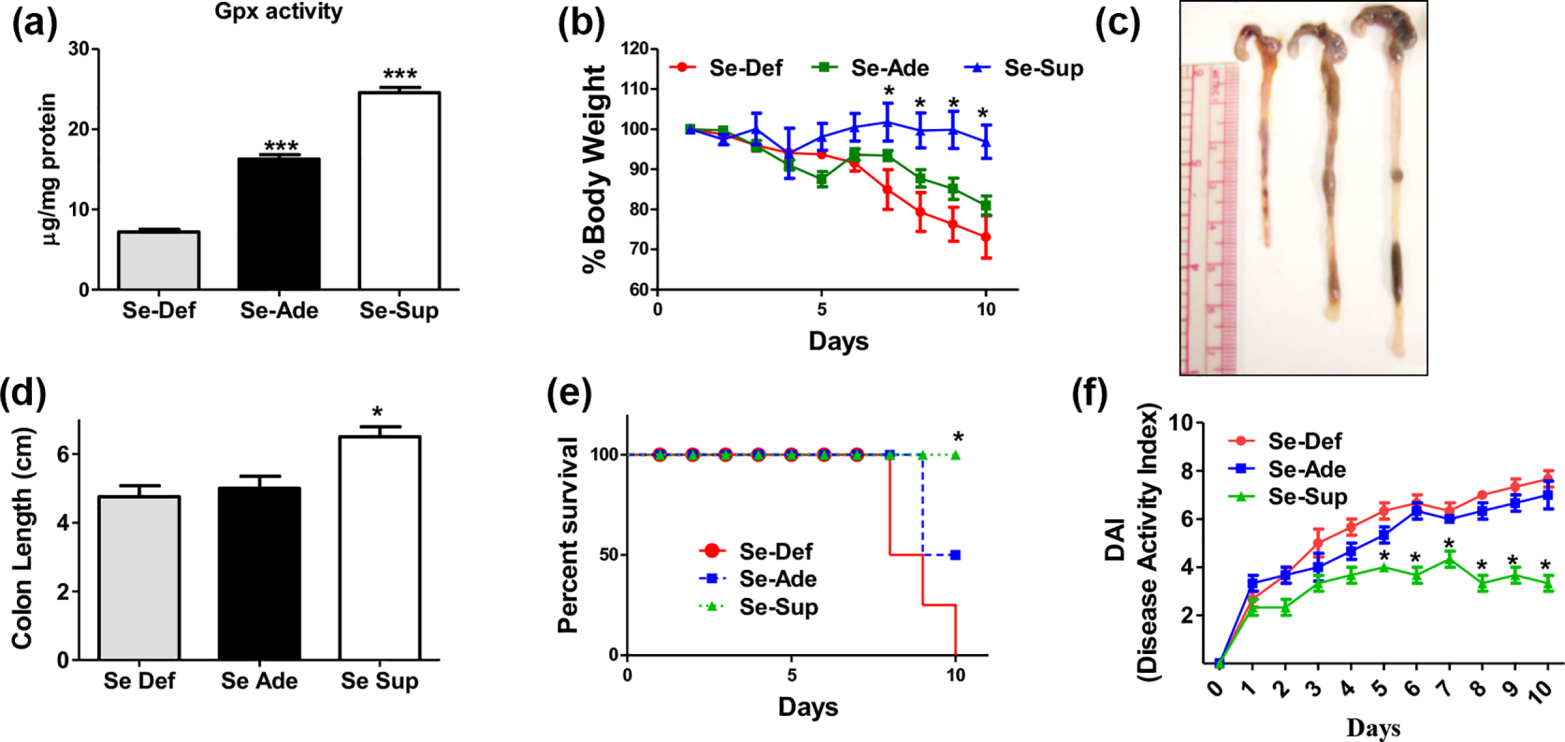
Se relives sign of inflammation in DSS colitis model. (a) Differential GPx activity in Se-Def, Se-Ade and Se-Sup. (b) Shows significant decrease in body weights in Se-Def group when compared with Se-Ade and Se-Sup group. (c, d) shortening of colons of Se-Def mice compared to Se-Ade and Se-Sup groups (e) Percent survival curves of three groups with Se-Def with poor survival when compared to Se-Ade and Se-Sup groups. (f) Disease activity index (DAI). Data is represented as mean ± SEM of atleast4 independent observations and ***, **, * represent p<0.001, p<0.01& p<0.05 respectively).

Expression and activity of selenoproteins such as GPx is used established marker of Se status in the body. Currently a dose dependent change in the activity and expression of dietary Se dependent glutathione peroxidase (GPx) was observed. The activity GPx was found to be significantly (p<0.001) decreased in Se-Def group compared to Se-Ade and Se-Sup groups (Fig 1A) indicating that differential physiological Se status has been achieved in these animals.

No statistically significant changes in the percent body weights of the animals with feeding diets containing differential levels of Se as sodium selenite was observed. However, following the induction of experimental colitis a significant decrease was seen in body weights as well as there is shortening of colon lengths of Se-Def group animals when compared to Se-Sup group (Fig 1B, 1C and 1D). While Se-Ade group animals also demonstrated some weight loss but the differences were not statistically significant. Further, the Kaplan–Meier survival curves demonstrated that the frequency by which mice in Se-Def groups succumb to experimental colitis was significantly higher compared to Se-Sup groups (Fig 1E). Consistent with these data Se-Sup mice demonstrated decreased disease activity index (DAI) compared to Se-Def and Se-Ade mice (Fig 1F).

**Fig 2:**
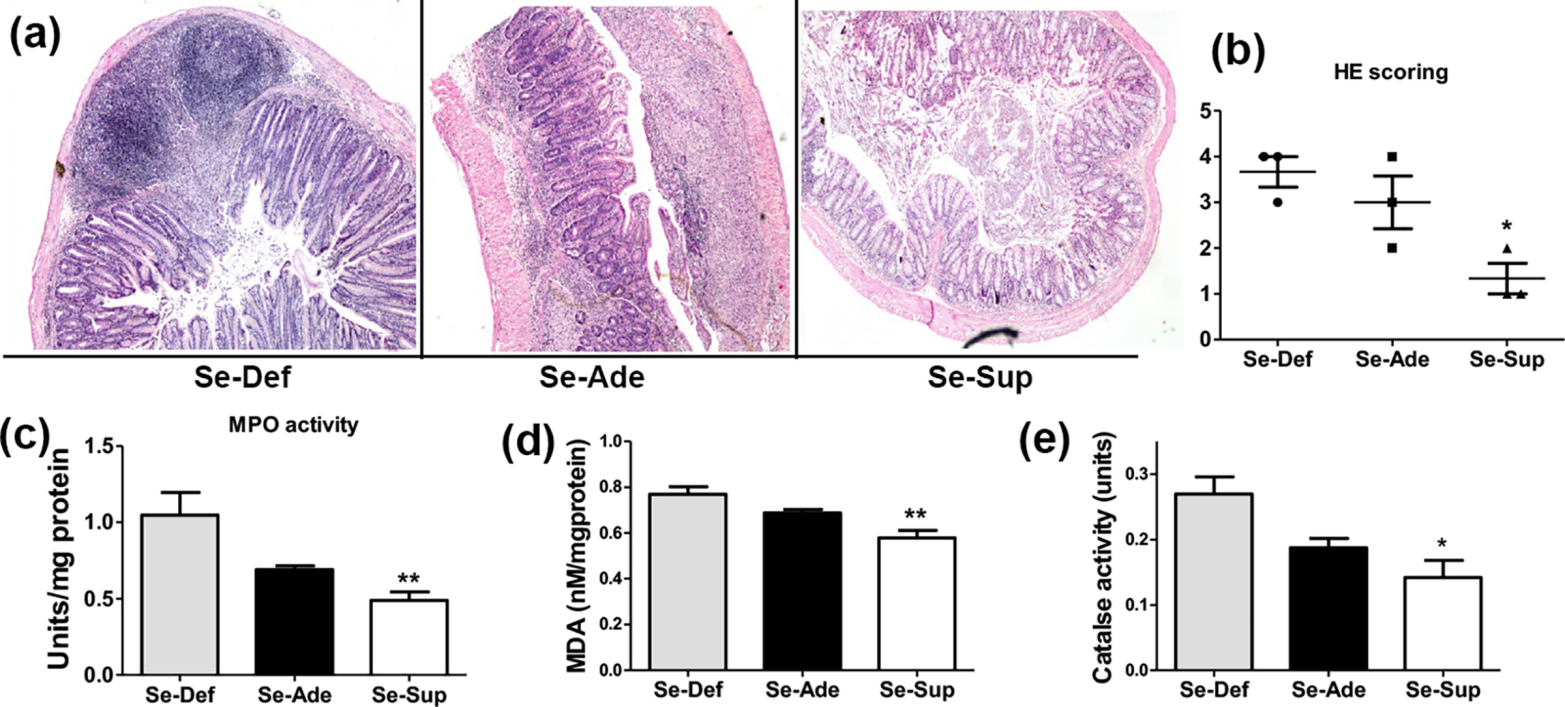
Redox mediated resolution of inflammation by Se. (a) and (b) shows histopathology images and scoring of colonic tissues in Se-Def, Se-Ade and Se-Sup groups. (c) Increased myeloperoxidase activity in Se-Ade and Se-Sup groups compared to Se Def groups. (d, e) Alteration in MDA levels and catalase activity after DSS treatment in colons of mice fed on diets containing different Se concentration. Data is represented as mean ± SEM of atleast4 independent observations and ***, **, * represent p<0.001, p<0.01& p<0.05 respectively.

Histopathological examinations of H/E stained colonic sections revealed that colon sections from Se-Def groups have increased neutrophil infiltration, mucodepletion, moderate to severe inflammation, cryptitis and crypt abscess (Fig 2A and 2B). Se-Ade mice showed sections of colon with partially denatured epithelial lining (Fig 2A and 2B) whereas DSS-treated Se-Sup group indicated negligible signs of mucosal damage. No evidence of cryptitis, crypt distortion, surface mucodepletion, metaplasia or dysplasia was found in Se-Sup group mice (y2A and 2B). Consistent with these finding increased MPO activity (p<0.01) in the colons of Se-Def mice indicated enhanced inflammation in contrast to Se-Ade and Se-Sup groups. This difference was statistically significant in Se-Sup group when compared to Se-Def groups (Fig 2C).

### Se ablates oxidative stress in experimental colitis

Plethora of pathophysiological properties of Se is owed to its antioxidant properties. Therefore, the changes in enzymatic and non-enzymatic indices of oxidative stress were measured in the colonic tissues of different groups. A highly significant increase (p<0.01) in lipid peroxidation (levels of MDA) and catalase activity was observed in Se-Def group as compared to Se-Ade and Se-Sup groups (Fig 2D and 2E).

### Modulatory effects of Se on intestinal marker enzymes

**Fig 3:**
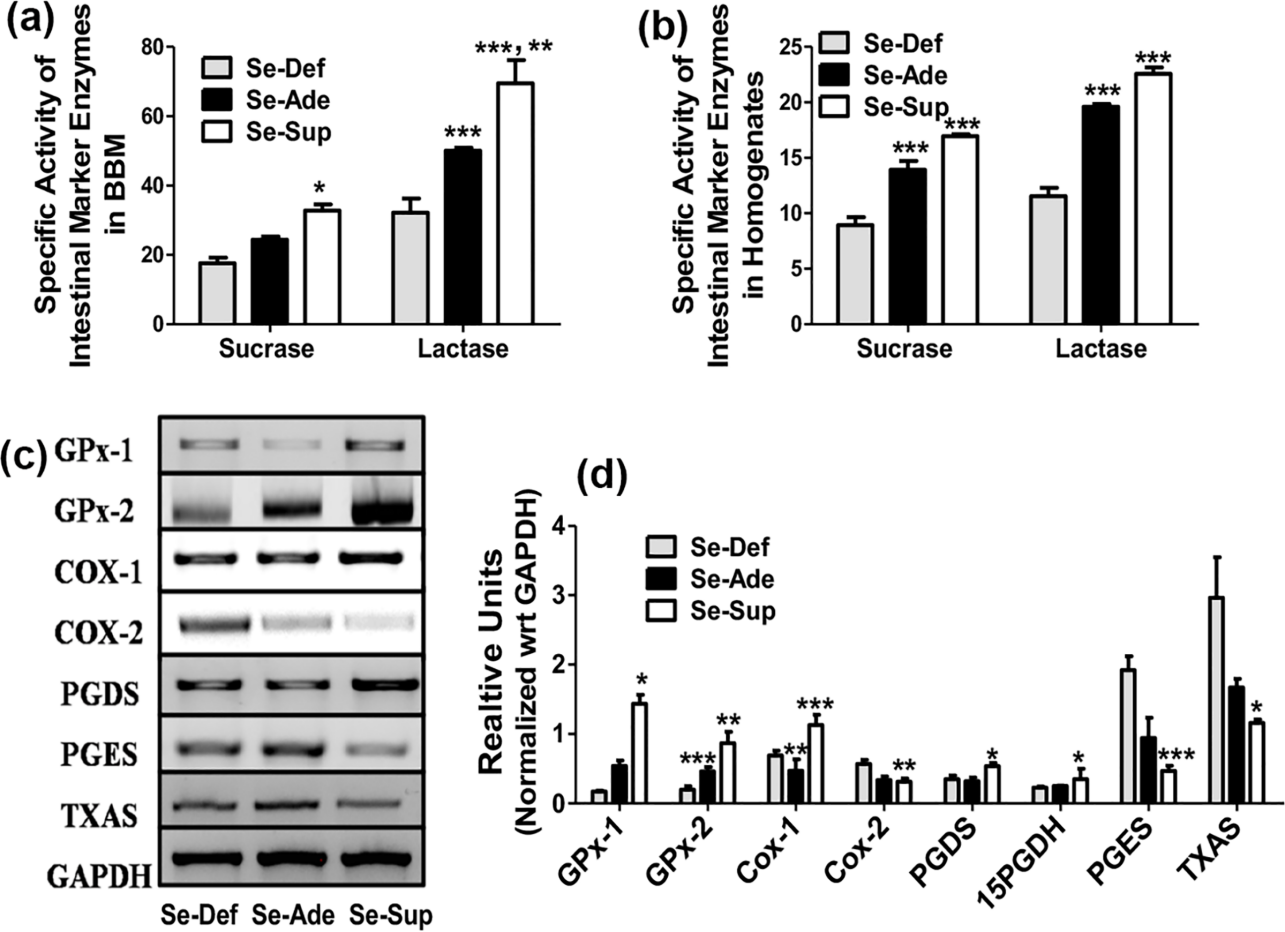
Protective effects of Se are mediated through modulation of Cox pathway. (a) and (b) represents the specific activity of intestinal marker disaccharidases in BBM and colon homogenates of Se-Def, Se-Ade and Se-Sup groups. (c) and (d) shows Se concentration dependent significant alterations in the mRNA and protein expressions of pro and anti-inflammatory markers. Data is represented as mean ± SEM of atleast4 independent observations and ***, **, * represent p<0.001, p<0.01& p<0.05 respectively.

Fig 3A and 3B demonstrate an alteration in the specific activities of intestine marker enzymes sucrase and lactase in Se-Def, Se-Ade and Se-Sup groups following DSS treatment. The debilitating effects of experimental colitis as observed through a significant decrease in intestinal disaccharidases (sucrase and lactase) were evident in Se-Def and Se-Ade groups compared to Se-Sup groups in both homogenate and BBM preparations of colons. However, no significant change in alkaline phosphatase activity was observed (data not shown). These findings are in corroboration with exacerbated inflammation seen under Se deficient conditions.

### Se modulates Cox mediated inflammatory pathway

Since, COX dependent inflammatory pathway is central to UC, changes in the expression of various factors involved in colitis associated inflammation/resolution were studied. The expressions of inflammatory COX-2, PGES and TXAS were significantly elevated in Se-Def group compared to Se-Sup indicating induction and resolution of inflammation under both conditions respectively. In contrast 15-PGDH, GPx-1/2 and hPGDS were increased in Se-Sup group compared to Se-Def and Se-Ade groups suggesting that Se at different dietary concentrations differentially regulates the inflammation in experimental colitis (Fig 3C and 3D).

### Se modulates biophysical parameters in experimental colitis

**Fig 4:**
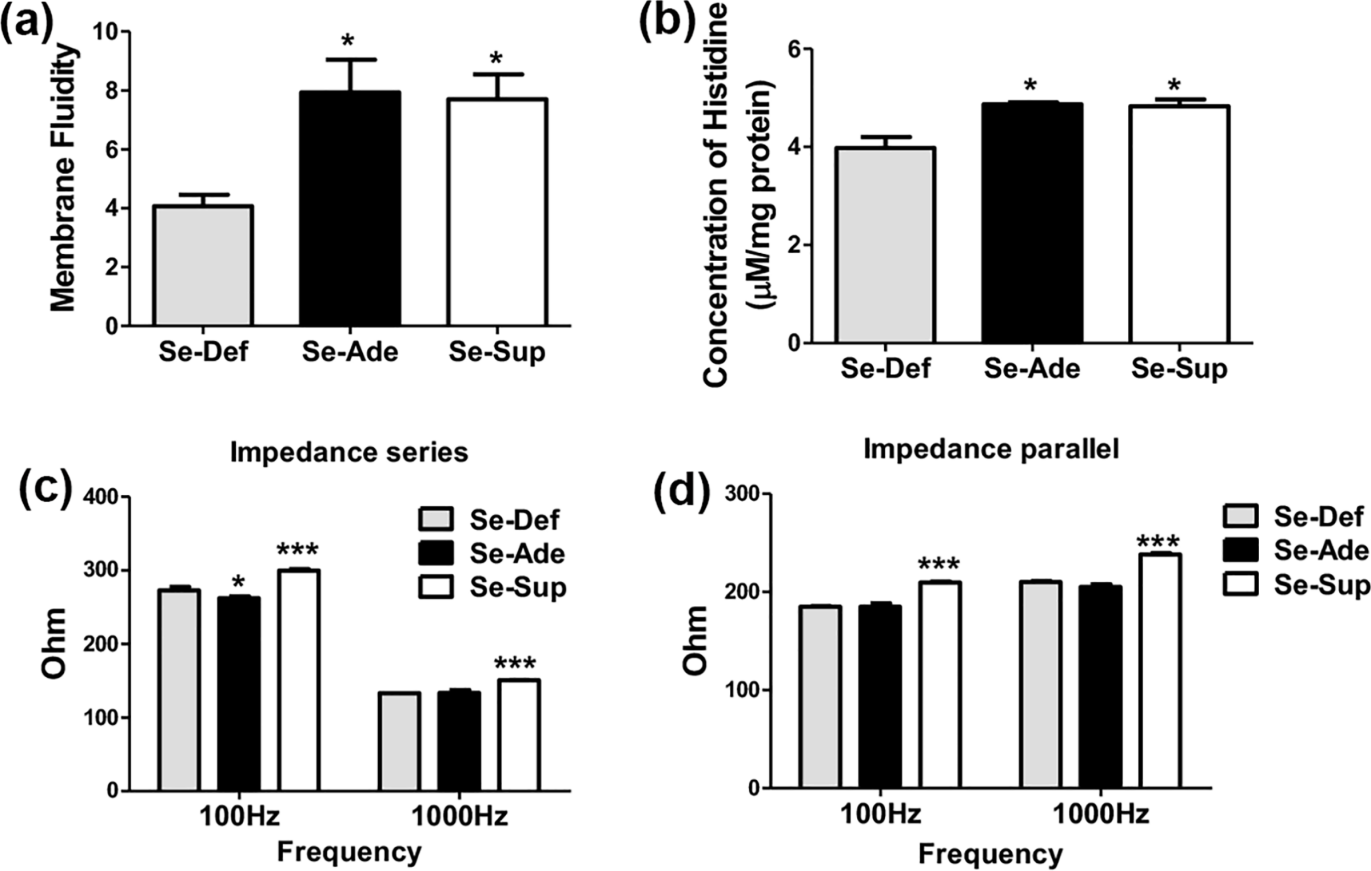
Biophysical characterization of protective effects of Se. (a) Demonstrates significant decrease in membrane fluidity in Se-D group in comparison with Se-Ade and Se-Sup groups (b) shows changes in the Histidine transport levels across the epithelial membrane (c, d) shows significant decrease in impedance at 1KHz and 100Hz in Se-Def group in comparison with Se-Ade and Se-Sup groups in series and parallel respectively. Data is represented as mean ± SEM of atleast 4 independent observations and ***, **, * represent p<0.001, p<0.01 & p<0.05.

Currently, transport of an amino acid (Histidine) across the membrane of the epithelial barrier was studied. Transport of histidine was found to be significantly decreased in the Se-Def group mice compared to Se-Sup groups (Fig 4B). These findings are in consistence with changes in the colonic membrane microviscosity or fluidity in which, a significant decrease(p<0.5) in the membrane fluidity was observed in mice of Se-Def group compared to Se-Sup and Se-Ade fed groups (Fig 4A).

Since, alterations or impairment in the intestinal structures and barrier function can change the epithelial membrane resistance, changes if any in the impedance (based on measurement of resistance) was used as surrogate markers to investigate the extent of epithelial damage following induction of colitis and protective effects of Se. Frequency ranges of 100 Hz and 1 KHz were used, both in parallel and series setup. A significant decrease in impedance in Se-D group at both 100 Hz and 1 KHz was observed compared to trends seen across colons of Se-S mice where a highly significant (p<0.001) increase in the impedance of epithelial layer was observed (Fig 4C and 4D).

**Fig 5:**
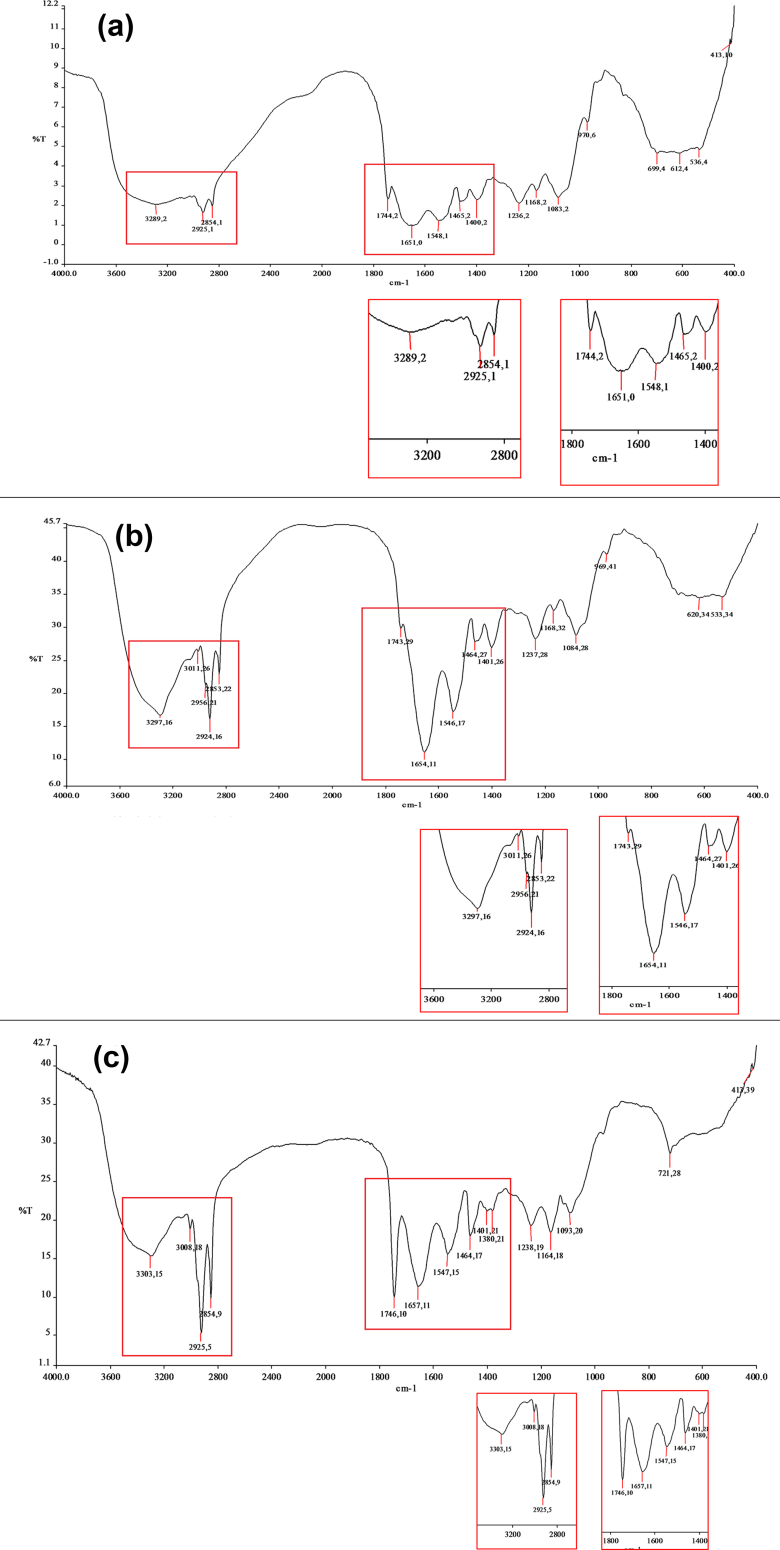
a, b and c represent FTIR spectra of colon in Se-Def, Se-Ade and Se-Sup groups respectively.

Currently, the FTIR spectra reflects the changes in the physical characteristics of colitis induced changes in the colonic mucosa of mice fed on diets containing different levels of Se (Fig 5A–5C). Table 2 shows a detailed comparative account of the different peaks and identified groups in the particular wave number in the colons of the Se-Def, Se-Ade and Se-Sup groups along with description of some of the spectral features. Not only a change in the number of peaks but also a shift in the wave numbers and peak heights corresponding to different vibrational frequencies has also been observed in DSS treated mice and naïve mice (Fig 5A–5C and a, b, c figures in S1 Fig).

**Table 2:** Comparative account of the different peaks and identified groups in the colons of the Se-Def, Se-Ade and Se-Sup groups along with description of the some of the spectral features of FTIR.

Wavenumber (cm^-1^)	wavenumber	wavenumber	Peak height Se-Def	Peak height Se-Ade	Peak height Se-Sup	Vibrational bond	Biomolecules involved
**Se-Def**	**Se-Ade**	**Se-Sup**					
536	533		4	18			
612	620		4	18			
699	700	721	4	18	28		
970	969		6	25		C-O stretch	DNA
1083	1084	1093	2	14	20	V_as_ PO^2-^ stretch	Nucleic acids
1168	1169	1164	2	17	18	C-O stretch	carbohydrates
1236	1237	1238	2	13	19	C-N stretch	N-H bend
1400	1402	1401	2	12	21	C-H bend	Amino acids
1465	1464	1464	2	12	17	C-H bend	Amino acids
1548	1546	1547	1	6	15	C-H stretch N-H bend	Amide II
1651	1652	1657	9	2	11	C = O stretch N-H bend	Amide I
1744	1743	1746	2	14	10		Phospholipids
2854	2853	2854	2	9	9	Terminal O-H, C-H, N-H	Cholesterol,creatine
2925	2924	2925	1	5	6	Terminal O-H, C-H, N-H	Cholesterol,creatine
	3011	3008		11	18	Terminal O-H, C-H, N-H	Cholesterol,creatine
3289	3298	3303	2	5	5	Terminal O-H, C-H, N-H	Cholesterol,creatine

## Discussion

Recently reviews on role of micronutrient Se in IBD have concluded that Se status and adequate dietary Se supply are of significant importance to colonic inflammation [42], [43]. Se is required at the active site of the enzyme GPx [44] and thus is considered a representative of availability of this metal in the body. However, studies suggest a great variance in the biological activity and toxicity of different inorganic and organic forms of selenium leading to conflicting results in its toxicity profiles [45]. Further, the route of administration and duration of administration adversely affect its absorption and bioavailability and thus wide spectra of pathophysiological effects. Despite these conflicts it remains evident that Se supplementation possesses anti-carcinogenic and anti-inflammatory properties and these effects are mediated through regulation of selenoproteins. In reference to colitis recent studies by Sang et al (2016, 2017) and Zhu et al (2017) have shown that different doses, duration and forms of Se have protective as well as therapeutic anti-inflammatory effects [14][15][16].

Currently Se concentration dependent increased activity and expression of glutathione peroxidase (GPx) is used a surrogate marker to establish physiological levels of Se as routinely used in literature [46], [25]. It was observed that dietary Se moderated the classical gross hallmarks of colitis in terms of body weights, colon lengths, DAI, and histopathological parameters which are indicators for the severity of the disease [27], [47]. Se-Def animals readily loose body weights, had shortened colons, and succumb to DSS induced inflammation. Concurrently, permanent tissue mounts of colons of Se-Def mice showed signs of chronic mucosal inflammation along with epithelial damage [25] and MPO activity which is an indicative of neutrophil infiltration [47]. Supporting epidemiological evidence also indicate inverse relationship between Se levels and incidence of UC in patients [20], [21], [22].

Since, COX dependent inflammatory pathway is central to UC, changes in the expression of various factors involved in colitis associated inflammation/resolution were studied. Increased levels of COX-2 and pro-inflammatory genes such as PGES and TXAS as seen under Se-Def conditions in fact validate above findings indicating deficiency induced acute inflammatory response [48] and severe tissue damages as seen in UC [49]. On the other hand, decrease in levels of these pro-inflammatory markers and concomitant increases in anti-inflammatory PGDS, 15PGDH and COX-1 suggest that Se by setting a favorable redox tone has the capability to resolve the inflammation *via* modulation of PG biosynthetic pathways [50]. A redox sensitive link modulating PGs metabolism in UC has been reported previously which can explain Se dependent regulation of the redox state of cells through reduction of RONS [51]. These findings are in agreement with previous reports suggesting that Se in a redox regulatory manner promotes resolution by PG class switching [25].

Redox status being important for the mucosal integrity, luminal nutrient absorption, mucus fluidity, and diverse microbiota, its alteration are clearly implied to affect the biophysical properties including transport, conductivity and membrane fluidity [52]. Currently, decrease in lipid peroxidation and Catalase in Se-Sup animals shows similar pattern as shown in membrane fluidity suggesting an inverse relationship between oxidative stress (OS) in UC and overall decrease in membrane fluidity as reported earlier [53]. Thus, decreased colonic membrane fluidity in Se-Def might be a cumulative outcome of oxidative stress and altered gut microbiota, which can deplete membranes of the lipids [52], [54]. These results can also be correlated to decreased GPx leading to accumulation of peroxides inside the cells and enhanced lipid damage [55], [56]. Contrary to this, in Se-Sup groups, Se as antioxidant selenoproteins such as GPx by reducing H_2_O_2_, lipid and phospholipid hydroperoxides [57] can prevent the attenuation of membrane fluidity. Additionally, altered junction structures and fluidity contributes to the barrier defect culminating in increased back leak, and reduced net ion transport [41]. This explains the decreased transport of histidine across the colonic epithelium in Se-Def mice compared to supplemented groups showing that ion transport is directly proportional to membrane fluidity. Additionally, altered junction structures and fluidity contributes to the barrier defect culminating in increased back leak, and reduced net ion transport [41]. This explains the decreased transport of histidine across the colonic epithelium in Se-Def mice compared to Se-Sup groups showing that ion transport is directly proportional to membrane fluidity. These debilitating effects are also evident through a significant decrease in intestinal disaccharidases (sucrase and lactase) [58].

Since, alterations or impairment in the intestinal structures and barrier function can change the epithelial membrane resistance, changes if any in the impedance was used as proxy markers to investigate the extent of epithelial damage following induction of colitis and protective effects of Se. Decrease in impedance in Se-Def group suggests alteration or impairment in the intestinal structures, epithelial barrier function and increase in leakiness, leading to changes the epithelial membrane resistance as studied earlier [59]. Previous reports have also shown that in UC, when inflammation becomes more intensive, ulceration on the lumen of the colon makes them more conductive [60]. However, in Se-Sup conditions increased impedance of epithelial layer is reflective of maintenance of tight junctions and membrane fluidity, thus preventing the changes in the resistance of epithelial layer.

Concurrent with above results, the FTIR spectra corresponding to different vibrations provided clues about alteration not only in the tissue architecture and structure but also of the nutritional and energy requirement following malignancy. Overall the wavenumbers and the peak heights in the spectral range of 400 cm^-1^–4000 cm^-1^were altered as a function of Se in the inflammatory conditions. The intensities of the peaks such as C = O band near 1743 cm^*−*1^ and C-H-stretching vibration bands near 2465 cm^-1^ to 2963 cm^-1^, 2966 cm^*−*1^, 2927 cm^*−*1^, and 2858 cm^*−*1^ are assigned to the fat in tissues [61]. These peaks were decreased in Se-Def and Se-Ade group suggesting loss of phospholipids and fats from the colonic mucosa and suggesting increased fat consumption due to increased nutritional and energy requirement in the development of ulcers [61]. These findings are also in accordance with the changes in microviscosity and impedance across the colonic mucosa A pattern of high intensity changes was noticed when Se-Def, Se-Ade and Se-Sup groups were compared with their respective naïve controls (i.e. without DSS). However, the changes in intensities were higher in Se-A group and its control when compared with other groups signifying the normal structural features of the colon with adequate levels of Se. The FTIR spectra of Se-Def naïve control shows less changes in peak intensities signifying that there are some structural changes in Se-Def naïve control which may be because of the ill effects caused by Se deficiency. The spectral differences in Se-Sup group and its naïve control are very less signifying that even after DSS administration the Se-S group recovers from inflammation to a large extent. A slight change in intensities near 1643 cm^*−*1^ corresponding to amide I and II band of protein and H–O–H deformation vibration of water was observed in Se-Def and Se-Ade groups when compared with Se-Sup, which has been previously used as an index of malignant colon tissues as compared to controls [61]. Studies have shown that the peak near 1460 cm^*−*1^ is stronger than or equal to that of 1400 cm^*−*1^ in the spectra of colitis samples. It was found that intensity of 1460 cm^-1^ peak decreased in case of Se-Sup group compared to Se-Def and Se-Ade evidently reflects the protective effects of Se supplementation against inflammatory colitis.

In conclusion, Se supplementation *via* its redox sensitive modulatory effects on the COX mediated PG biosynthetic pathway aids to resolve colitis associated inflammation. A progressive attempt and significant progress has been made to comprehend and correlate the molecular mechanism/s of inflammation in UC and their biophysical characterization. Extensive studies in future are warranted to understand and draw causative correlations behind these mechanisms with an aim to develop efficient methods to effectively resolve the inflammation seen in various inflammatory pathologies such as UC.

## Supporting information

S1 Checklist Plos One humane endpoint checklist. (DOCX)

S1 Fig a, b and c Figs represent the FTIR Spectra of the colons of the naïve (non DSS) mice from Se-Def, Se-Ade and Se-Sup controls respectively.
(TIF)
