# Supplementary figures and images for: Resolution of Cox mediated inflammation by Se supplementation in mouse experimental model of colitis
Source: PLoS One. 2018 Jul 31;13(7):e0201356. doi: 10.1371/journal.pone.0201356 (PMC6067745; doi:10.1371/journal.pone.0201356)

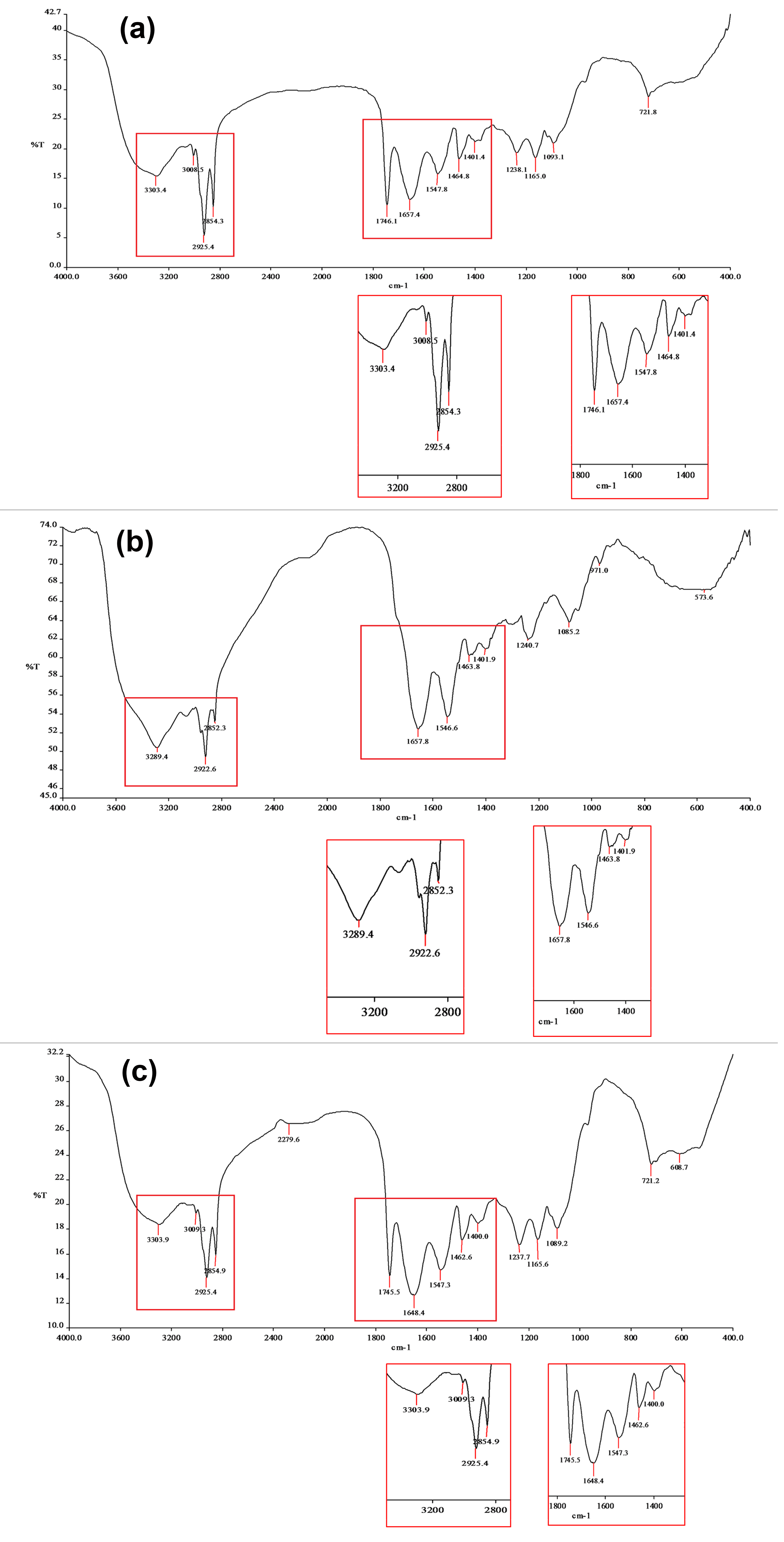

Supplement: S1 Fig — a, b and c Figs represent the FTIR Spectra of the colons of the naïve (non DSS) mice from Se-Def, Se-Ade and Se-Sup controls respectively. (TIF) [file pone.0201356.s002.tif]
